# Human cytomegalovirus glycoprotein polymorphisms and increasing viral load in AIDS patients

**DOI:** 10.1371/journal.pone.0176160

**Published:** 2017-05-03

**Authors:** Xiao-Jing Jiang, Jun Zhang, Yong Xiong, Gerhard Jahn, Hai-Rong Xiong, Zhan-Qiu Yang, Yuan-Yuan Liu

**Affiliations:** 1 Department of Infectious Diseases, Wuhan General Hospital of Guangzhou Military Command, Wuhan, Hubei, P. R. China; 2 Department of Emergency, Ganzhou People's Hospital, Ganzhou, Jiangxi, P. R. China; 3 Department of Infectious Diseases, Zhongnan Hospital of WuhanUniversity, Wuhan, Hubei, P. R. China; 4 Institute of Medical Virology and Epidemiology of Viral Diseases, University Hospital of Tübingen, Tübingen, Germany; 5 Institute of Medical Virology, School of Basic Medical Sciences, Wuhan University, Wuhan, Hubei, P. R. China; University of St Andrews, UNITED KINGDOM

## Abstract

**Background:**

Multiple strains infection of human cytomegalovirus (HCMV) was found to be correlated with increased viral load in immunodeficient patients. However, the pathogenic mechanism underlying this correlation remains unclear. To evaluate genetic polymorphisms of HCMV glycoprotein and their potential role in its viral load, HCMV glycoprotein B, N, and O (gB, gN and gO) genotypes was studied in the population of HCMV infected acquired immune deficiency syndrome (AIDS) patients. The association between glycoprotein polymorphisms and HCMV viral load was analyzed.

**Methods:**

The genetic polymorphisms of glycoprotein from sera of 60 HCMV infected AIDS patients was investigated by multiplex nested PCR and sequencing. HCMV viral load was evaluated by quantitative PCR.

**Results:**

gB1, gO1a, and gN4a were the predominant glycoprotein genotypes in HCMV infected AIDS patients and composed 86.96%, 78.8%, and 49.2%, respectively. Only gN4a genotype infection significantly increased viral load (*P* = 0.048). 71% (43/60) of HCMV infected AIDS patients were found to carry multiple HCMV strains infection. A novel potential linkage of gO1a/gN4a was identified from multiple HCMV infected patients. It was the most frequent occurrence, accounted for 51.5% in 33 patients with gO and gN genotypes infection. Furthermore, the gO1a/gN4a linkage was correlated to an increased viral load (*P* = 0.020).

**Conclusion:**

The gN4a correlates to higher level HCMV load in AIDS patients. Interestingly, a novel gO1a/gN4a linkage is identified from the patients with multiple HCMV strains infection and is also associated with an increased viral load. Therefore, the pathogenic mechanism underlying glycoprotein polymorphisms and interaction of variants should be analyzed further.

## Introduction

Human cytomegalovirus (HCMV), a member of the herpesvirus family, infects 50–100% of adults worldwide. The primary infection is often acquired during early childhood and later persists lifelong in a latent stage. It is usually asymptomatic in immunocompetent individuals infected with HCMV. However, reactivate or exogenous reinfection with HCMV may occur and become a major viral cause of morbidity and mortality in immunocompromised patients such as Acquired Immune Deficiency Syndrome (AIDS) patients and stem cell-transplant recipients.

In immunodeficient patients, the occurrence and severity of HCMV disease generally are often associated with the grade of immunosuppression. It also may correlate with host susceptibility factors and HCMV strain virulence. The difference of HCMV virulence between different HCMV strains is due to their genetic variants [1], such as their variation in HCMV encoded envelope glycoprotein B (gB), gM, gN, gH, gL, and gO. These glycoproteins are involved in virus entry, egress, transmission and are major targets of immune responses [1–6]. Different levels of variation of glycoprotein genes were observed in HCMV clinical isolates and laboratory adapted strains. Except that the gH and gM genes were highly conserved. The gN and gO genes have hypervariation locus, which account for 40–50% in the level of variation [7]. The gB and gH genes present a modest variation (5–11%) [8, 9].

According to the sequence variation of UL55 gene that encodes gB, there are four major gB genotypes(gB1, gB2, gB3, gB4) [10–13]. In addition, three nonprototypic genotypes (gB5, gB6, gB7) have been identified [14] [15]. Previous studies showed that gB2 is the predominant gB type in AIDS patients [11–13, 16, 17]. However, the association of gB genotypes and disease progression is still controversial. The UL73 encodes gN, which shows four genotypes (gN1, gN2, gN3, and gN4). The gN3 genotype includes two subgenotypes (gN3a and gN3b) and gN4 includes three subgenotypes (gN4a, gN4b, and gN4c) [7, 18, 19]. The UL74 encodes gO and five major genotypes (gO1, gO2, gO3, gO4 and gO5) with minor subtypes (gO1a, gO1b, gO1c, gO2a, gO2b) have been defined in the gO gene [10, 20]. Despite high variation locus at gO and gN genes, the biological meaning of the variation is largely unknown. Recently seven linkages between the gO and gN genotypes have been defined. They are gO1a/gN1, gO1b/gN3a, gO1c/gN4c, gO2a/gN3b, gO2b/gN2, gO3/g4a, and gO4/gN4b [21]. However, the effect of these gO/gN linkages on HCMV virulence is not clearly understood. gN forms a complex with gM (gM/gN) and gO forms a complex with gH/gL (gH/gL/gO), and these two complexes play an essential role in viral entry, transmission and exit. We hypothesize that there is interaction between the complex of gM/gN and gH/gL/gO, which may have impact on virulence of HCMV and is associated with clinical outcomes.

To assess the effect of HCMV glycoprotein polymorphisms on viral virulence, a cohort of Chinese AIDS patients were selected for the study. We investigated the distribution of gB, gO, and gN genotypes and their correlation with viral load in these patients. We aslo investigated the association between different gO/gN linkage groups and viral load.

## Materials and methods

### Patients and data collection

Blood samples were obtained from 106 hospitalized AIDS patients with CD4+ count < 100 cells/mm^3^ during the period of May 2012 to May 2014 at the department of infectious diseases, Zhongnan Hospital of Wuhan University, China. Ethical approval for the study was obtained from the Research Ethics Committee of Wuhan University. All the patients were informed the consent of the study and signed statement before starting the research work. The clinical data were registered, including gender, age, current HIV treatment, CD 4+ counts, and opportunistic infections (Table 1). All the patients had not received any antiherpesvirus treatment before they participated in this study.

**Table 1:** Characteristics of AIDS patients with and without detectable HCMV IE4 gene in serum samples.

Characteristics	HCMV positive (n = 65)	HCMV negative (n = 41)
Gender		
Male	47	31
Femal	18	10
Age (Mean±SD)	40.0±12.3	36.5±12.0
Current HIV treatment		
Yes	20	15
No	45	26
Mean CD4+ counts, cells/ mm^3^	17.0	27.0
Opportunistic infections		
Toxoplasmosis	1	1
Tuberculosis	16	49
Cryptococcosis	3	7
Pneumocystosis	4	1
fungal infection	31	15
HCMV retinitis	9	6
HCMV encephalitis	2	0
HCMV colitis	11	3

#### HCMV DNA isolation and detection

Sera from patients were used for DNA isolation.DNA was extracted from sera using AxyPrepTM body Fluid Viral DNA/RNA Miniprep Kit (Axygen, USA). The nested PCR was used to amplify HCMV IE4 gene in the sera samples. The primers for amplification and PCR procedure were identical to our previous study [22]. Samples determined to be HCMV DNA positive were further examined for genotyping and viral load.

The real-time quantitative PCR with SYBR GREEN (Toyobo, Japan) was carried out for detection of HCMV DNA load as described previously [23]. The primers for quantitative PCR were designed by Premier 5.0 software and listed in Table 2. The CT values obtained for the samples were plotted on the standard curve and then the copy number was calculated. The HCMV DNAs were defined as negative when the CT values exceeded 40 cycles.

**Table 2:** Primers used for multiplex nested PCR, real-time PCR and gN sequencing.

Gee	Method	Primer	Primer sequence (5’ to 3’)	GenBank accession	Position	Product length
gB	Primary PCR	UL55 up	TTTGGAGAAAACGCCGAC	KP745727.1	83930 to 83947	751
		UL55 low	CGCGCGGCAATCGGTTTGTTGTA	KP745727.1	83196 to 83218	
	Nested PCR	gB1	ATGACCGCCACTTTCTTATC	KF297339.1	84086 to 84105	420
		gB2	TTCCGACTTTGGAAGACCCAACG	FJ527563.1	83509 to 83531	613
		gB3	TAGCTCCGGTGTGAACTCC	GU937742.1	83097 to 83115	190
		gB4	ACCATTCGTTCCGAAGCCGAGGAGTCA	GU179291.1	83557 to 83579	465
		gB5	TACCCTATCGCTGGAGAAC	AF062421.1	78 to 96	139
		gB low	GTTGATCCACRCACCAGGC	KP745727.1	83284 to 83302	
gN	Primary PCR	UL73 up	AGTCGATTCGGTCGGTCAAC	KP745723.1	106325 to 106344	469
		UL73 low	CCACCCTCAATAGCCTTTGGT	KP745723.1	106767 to 106787	
	Nested PCR	gN 1	TTCTGCTAGCGTATCAACTACC	FJ527563.1	106796 to 106817	283
		gN 2	AGTGCAAAACACTGGTGCT	GU179291.1	106907 to 106925	380
		gN 3b	CACAACCACATTAACGAGT	X512202.1	106738 to 106756	214
		gN 4a	CAACAATACGTCGACTGCTAGCACAC	AY446894.2	107107 to 107132	325
		gN 4b/c	GACAACTAGTACAACTACGGTGACAA	FJ616285.1	106421 to 106446	244
		gN low	GACATTGCTGCTTCCAGAA	KP745723.1	106732 to 106750	
gO	Primary PCR	UL74 up	TGGTGTGATGGAGTGGAAC	KP745727.1	107053 to 107071	1901
		UL74 low	AACGGTAGATGAGCAGCAAAACGAC	KP745727.1	108930 to 108954	
	Nested PCR (Group 1)	gOup-1	GCTCATGGCGTTAACCAGGTA	KP745727.1	108144 to 108164	
		gO1a-2	ATAATCGTCCTTGGAGGGGC	FJ527563.1	108349 to 108368	582
		gO 2a	ACTGTTAATATGACCGAGTTTCCT	EU348354.1	244 to 267	441
		gO 3	TCAAACAGGCCGAAAGATGA	AF531318.1	393 to 412	304
		gO 4	ACTGCAAGAACTAGCGTCAA	FJ616285.1	107859 to 107878	520
		gO 5	CAGTACTCAACTCCGAAAACCA	AY446894.2	108468 to 108489	352
	Nested PCR (Group 2)	gO up-2:	GCTCATGGCGTTAACCAGG	KP745727.1	108144 to 108162	
		gO 1a-1	TAATCGTCCTTGGAGGGGCT	FJ527563.1	108348 to 108367	581
		gO 1b	GTAGGGCTGCGGTAAGATTAT	AF531354.1	86 to 106	608
		gO 1c	AGGCAACACGTTAAAAATCTTACT	KF297339.1	108979 to 109002	462
		gO 2b	ATAATCAAACGGCTCAGAAAATCA	GU179288.1	108357 to 108380	308
IE	real-time PCR	P1	5’-TTTAGCACGGGCCTTAGCCT-3’	FJ527563.1	172849 to 172868	76
		P2	5’-GCTGCATGATGTGAGCAAGGG-3’		172898 to 172918	
gN	sequencing	gN up	5’-TGGTGTGTGATGGAGTGGAAC-3’	KP745722.1	106845 to 106861	417
		gN low	5’-TAGCCTTTGGTGGTGGTTGC-3’		107243 to 107262	

* The multiplex nested PCR assay was performed for genotyping. The first PCR round was designed to amplify the segment containing the major variable region of target gene. The primers for primary PCR were designed on the conserved regions. The nested PCR round was carry out with a set of genotype-specific primers. For gB/gN genotyping, the multiplex nested PCR was performed using a set of genotype-specificprimers (gB1, gB2, gB3, gB4, gB5/ gN1, gN2, gN3b, gN4a, gN4b/c) and a single common primer (gB low/ gN low), respectively. For detection of gO genotypes, the multiplex nested PCR was divided into 2 groups. The gO up-1 was used as the common primer and a set of genotype-specific primers (gO1a-2, gO2a, gO3, gO4, gO5) was used to distinguish five gO genotypes in group1. In group2, the gO up-2 was used as the common primer and gO1a-1, gO1b, gO1c, gO2b were used as the genotype-specificprimers.

### Genotyping

For the HCMV DNA positive samples, the multiplex nested PCR assay was used for gB, gN and gO genotyping. The primers are listed in Table 2. For gB and gN genotyping, the PCR assays were operated as described by Tarragó [17] and Pignatelli [24]. The gN genotyping results were further confirmed by PCR and sequencing. For gO genotyping, due to the complexity of the gO genotype, the multiplex PCR was divided into 2 groups. The one group was designed to distinguish five gO genetypes (gO1a, gO2a, gO3, gO4, gO5), the other one was to genotype gO1a, gO1b, gO1c, gO2b. The first PCR round condition was 95°C for 5min and 34 cycles of 94°C for 1min, 57°C for 1min, 72°C for 1min, and a final elongation at 72°C for 10 min. In the second round, the annealing temperature of the nest PCR for group 1 and group 2 was 58°C, others conditions were as same as the first round. All the reactions of PCR were performed in 50μl volume containing 25μl of Premix Tag (TAKARA), 5 μl DNA, 20pmol primers and H_2_O. HCMV DNA sample from HCMV AD 169 strain was used as positive control and distilled water was used as negative control. Then the gB, gN and gO genotypes were differentiated by agarose gel electrophoresis.

### DNA sequencing and sequences analysis

Genotyping of gN was also achieved by DNA sequencing. The products of PCR were purified (QIAquick PCR Purification Kit, Qiagen) and sequenced by Sangon Biotech (Shanghai, China) Co.Ltd. The primers are listed in Table 2. Data were assembled manually and edited by ClustalX 2.0 and MEGA 5.1 software.

### Statistical analyses

All statistical analysis was performed using SPSS Statistics 20.0 (IBM, USA). Continuous variables were compared between 2 groups by the independent samples t-test for normally distributed or Mann-Whitney U-test for non-normally distributed variables. Two-tailed P values of below 0.05 were considered significant.

## Results

### Frequency of mixed HCMV infection with multiple gB, gO and gN genotypes in AIDS patients

The quantitative PCR was conducted to detect HCMV DNA from blood samples of 106 patients with AIDS. 61.3% (65/106) of patients were HCMV DNA positive. Only 60 out of these 65 HCMV infected AIDS patients had gB, gO or gN genotyping data. 71% (43/60) of patients were found to carry mixed infections with multiple genotypes in at least one genetic locus of gB, gO and gN.

### Distribution of gB, gO and gN genotypes in HCMV infected AIDS patients

**Fig 1:**
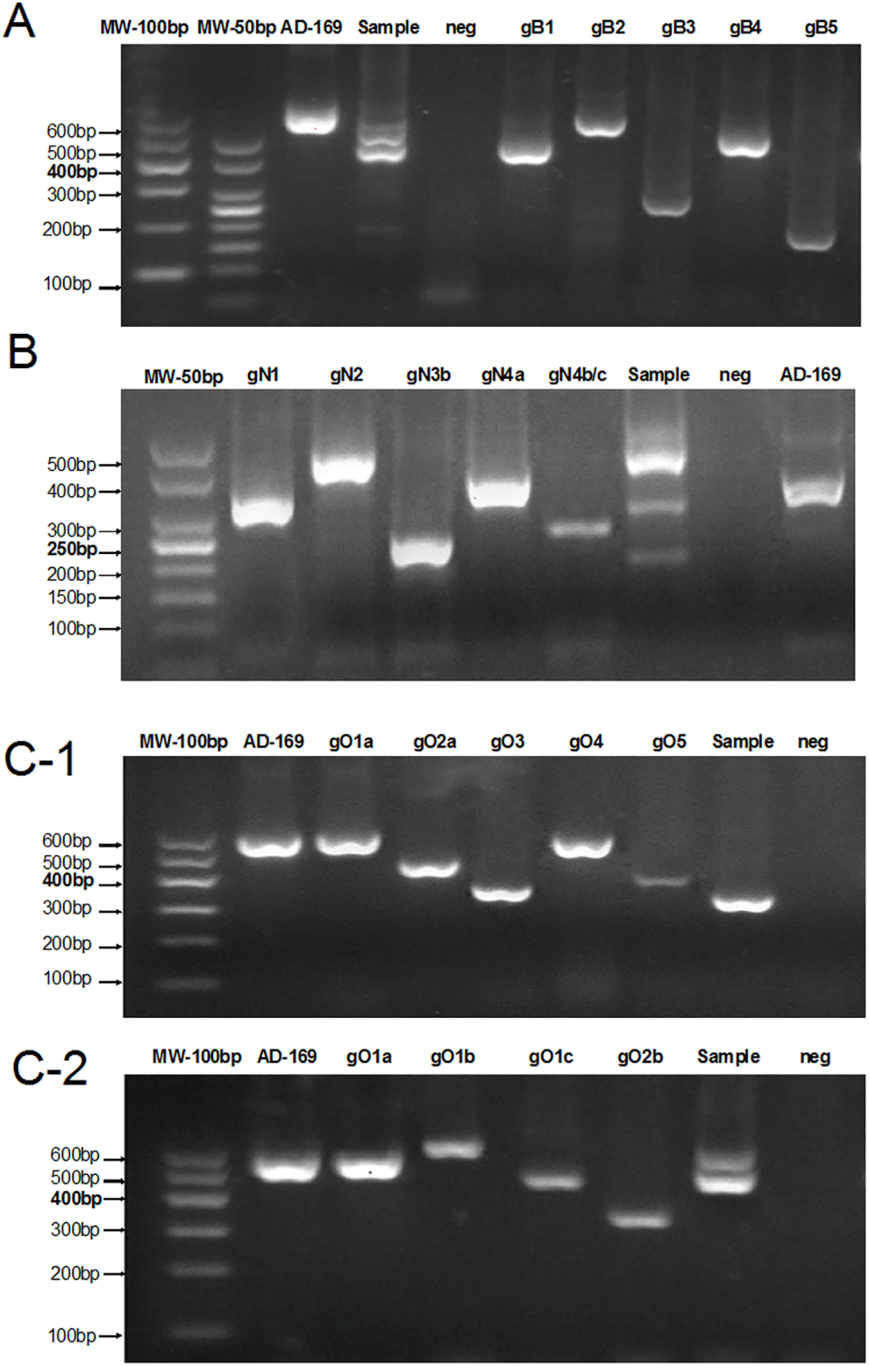
Multiplex nested PCR analysis for identification of HCMV gB, gN, gO genotypes (AD 169 is HCMV laboratory strain). A) Multiplex nested PCR analysis for HCMV gB genotyping. Size: gB1, 420bp; gB2, 613bp; gB3, 190bp; gB4, 465bp; gB5, 139bp. AD-169 (gB2); Sample (gB1+ gB3+gB4). B) Multiplex nested PCR analysis for HCMV gN genotyping. Size: gN1, 283bp; gN2, 380bp; gN3 214bp; gN4a, 325bp, gN4b/c, 244bp. AD-169 (gN1); Sample (gN1+gN2+gN3b). C-1) Multiplex nested PCR analysis for HCMV gO genotyping (Group1). Size: gO1a, 582bp; gO2a, 441bp; gO3 304bp; gO4, 520bp; gO5, 352bp. AD-169 (gO1a); Sample (gO3). C-2) Multiplex nested PCR analysis for HCMV gO genotyping (Group2) Size: gO group2: gO1a, 581bp; gO1b, 608bp; gO1c, 462bp; gO2b, 308bp. AD-169 (gO1a); Sample (gO1a+gO1c).

The gel electrophoresis of multiplex nested PCR products was performed for distinguishing genotypes of gB, gO and gN (Fig 1, S1 Table).

The gB genotype was successfully determined from 76.7% (46/60) of HCMV infected AIDS patients. The genotype gB1 was predominant and composed 87.0% (40/46) of patients, followed by gB3 in 60.9% (28/46), gB2 in 19.6% (9/46), gB4 in 17.4% (8/46), gB5 in 13.0% (6/46).

The gO genotype was determined from 55% (33/60) of HCMV infected AIDS patients. The gO1a was predominant and composed 78.8% (26/33) of patients, followed by gO3 in 39.4% (13/33), gO2b in 24.2% (8/33), gO1c in 15.2% (5/33), gO2a and gO5 in 12.1% (4/33), respectively, and gO1b in 3.0% (1/33).

The gN genotype was determined from 98.3% (59/60) of HCMV infected AIDS patients. The gN4a was predominant and accounted for 49.2% (29/59) of patients, followed by gN2 in 45.8% (27/59), gN1 in 28.8% (17/59), gN3b in 23.7% (14/59), gN4b in 10.2% (6/59), gN3a in 8.5% (5/59), and gN4c in 5.1% (3/59).

### Analysis of predominant glycoprotein genotypes and viral load in HCMV infected AIDS patients

**Fig 2:**
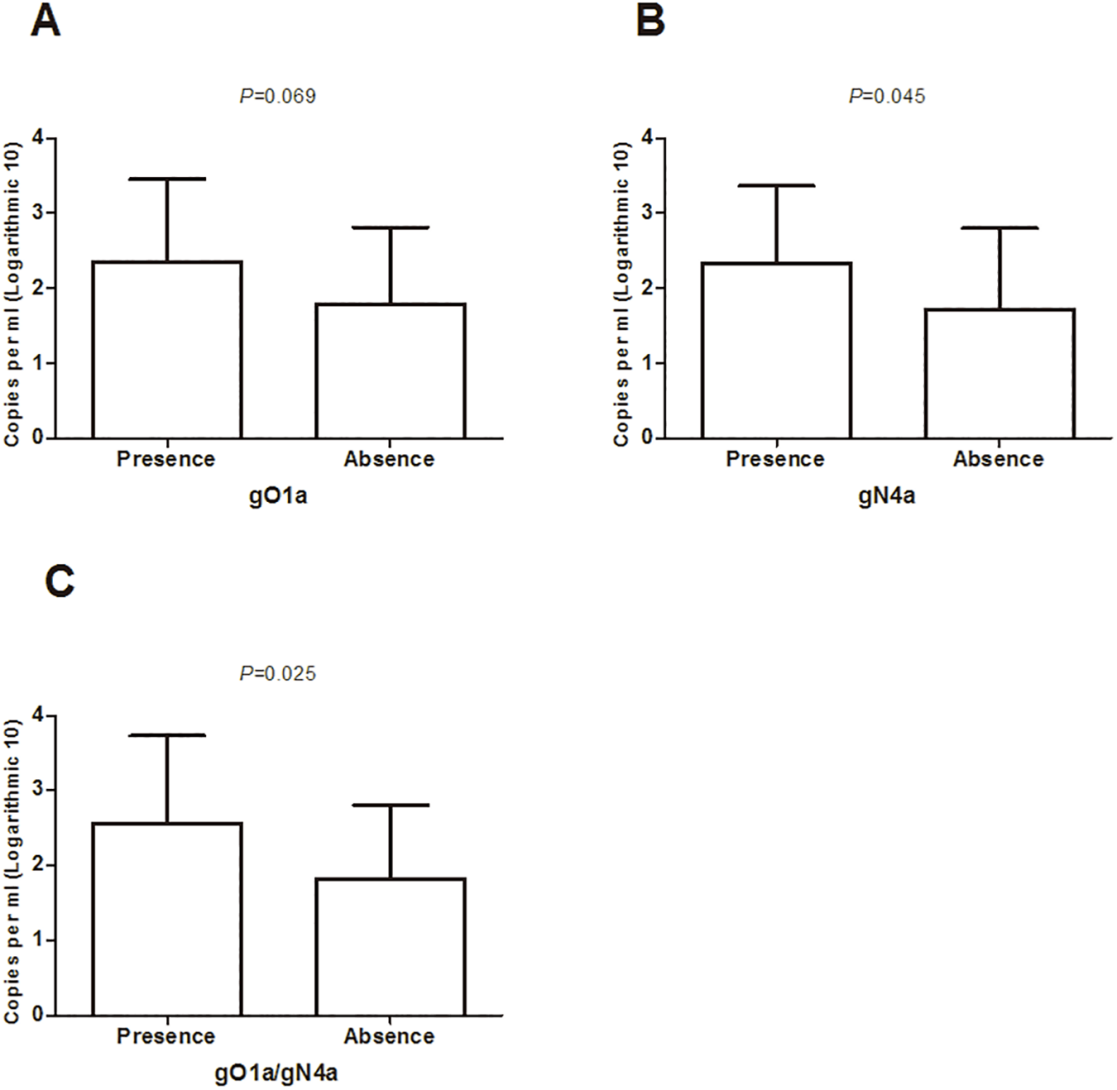
Comparison of HCMV viral load as measured by real-time quantitative PCR in blood samples and distribution of glycoprotein genotypes gO and gN genotypes in AIDS patients. A) The difference between the viral load within groups presence and absence of gO1a was not statistically significant (P = 0.062); B) The difference between the viral load within groups with and without gN4a was statistically significant (P = 0.048); C) The difference between the viral load within groups presence and absence of gO1a and gN4a was statistically significant (P = 0.020).

Comparison of viral load between the groups with and without gB1 infection showed that no significant difference was observed. In addition, no significant difference was found regarding viral load between groups with and without gO1a infection. Interestingly, the viral load of patients with gN4a infection was significantly higher than patients without gN4a infection (*P* = 0.048). Other genotypes were also analyzed, but no significant difference was found regarding to viral load. Data are shown in Fig 2A and 2B, Table 3 and S1 Table.

**Table 3:** HCMV viral load in patients with presence and absence of gO1a and gN4a genotype infection.

	gO1a(n)			gN4a(n)		
	Presence (24)	Absence (26)	*P* Value	Presence (27)	Absence (23)	*P* Value
Viral load (log10 copies/ml)	2.4(1.8–3.0)	1.9(1.3–2.6)	0.062	2.4(1.7–3.0)	1.9(1.2–2.5)	0.048

Values are given as median (interquartile range).

### Analysis of genetic linkage of gO/gN variants in multiple HCMV strains infected AIDS patients

**Fig 3:**
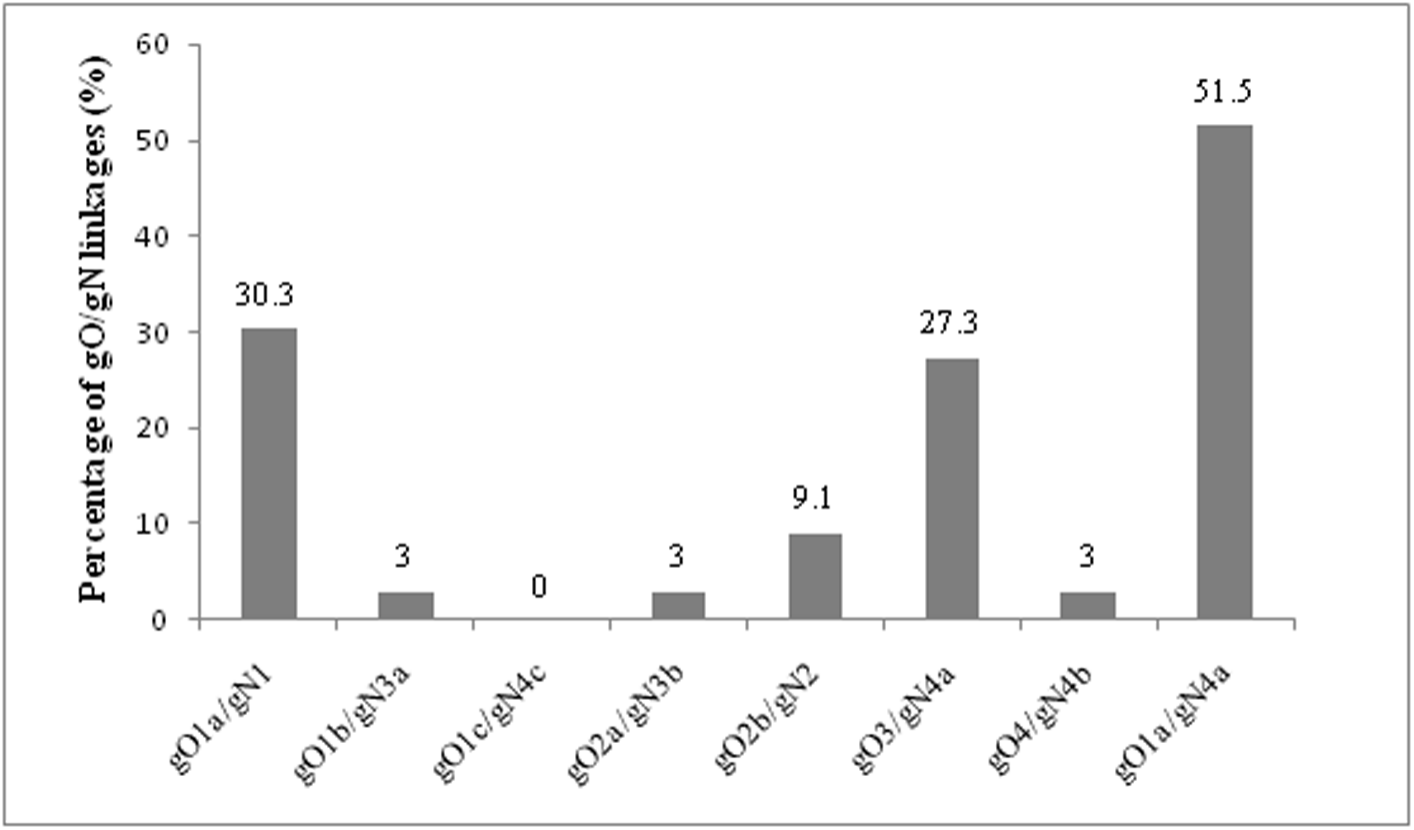
Distribution of different gO/gN linkages in AIDS patients with multiple HCMV genotypes infection.

Both gO and gN genotypes were identified from 33 patients with multiple HCMV strains infected AIDS patients. In the investigated sera, only 5 patients with single genotype in both gO and gN infection were found. Two sera samples contained indentified genetic linkage of gO1a/gN1 and the other three samples contained gO1a/gN4a, gO1a/gN3b and gO1a/gN2. Sera of 28 patients contained multiple genotypes in at least one genetic locus of gO and gN. The association of gO1a and gN4a was the most frequent appearance in all these samples and accounted for 51.5% (17/33). The linkage of gO1a/gN4a was a new observation in this study and has not been described previously [21]. Other associations of gO and gN were observed as gO1a/gN1 in 30.3% (10/33), gO3/gN4a in 27.3% (9/33), gO2b/gN2 in 9.1% (3/33), gO1b/gN3a, gO2a/gN3b, and gO4/gN4b in 3.0% (1/33), respectively. The percentage of all association of gO and gN in multiple HCMV strains infection patients is shown in Table 4 and Fig 3.

**Table 4:** Association of gO and gN genotypes in multiple HCMV infection.

gO									
gN		1a(n = 26)	1b(n = 1)	1c(n = 5)	2a(n = 4)	2b(n = 8)	3(n = 13)	4(n = 2)	5(n = 4)
	1(n = 11)	**10(30.3)**	0(0)	1(3.0)	1(3.0)	3(9.1)	4(12.1)	1(3.0)	0(0)
	2(n = 17)	13(39.4)	1(3.0)	4(12.1)	1(3.0)	**3(9.1)**	8(24.2)	0(0)	3(9.1)
	3a(n = 2)	1(3.0)	**1(3.0)**	1(3.0)	1(3.0)	0(0)	1(3.0)	0(0)	1(3.0)
	3b(n = 6)	3(9.1)	0(0)	0(0)	**1(3.0)**	2(6.1)	3(9.1)	0(0)	0(0)
	4a(n = 21)	**17(51.5)**	1(3.0)	3(9.1)	3(9.1)	4(12.1)	**9(27.3)**	2(6.1)	4(12.1)
	4b(n = 4)	4(12.1)	0(0)	0(0)	1(3.0)	2(6.1)	0(0)	**1(3.0)**	0(0)
	4c(n = 2)	1(3.0)	0(0)	**0(0)**	0(0)	0(0)	1(3.0)	0(0)	0(0)

Data are expressed as number (%) of patients.

### Analysis of gO/gN genotype and viral load

In order to study the effects of gO/gN genotype linkages on HCMV viral load, the independent samples t-test was performed. The linkages of the certain gO/gN genotypes (gO1a/gN1 and gO3/gN4a) were not associated with the viral load. However, the presence of gO1a/gN4a linkage group showed a significant higher viral load compared with the absence of the gO1a/gN4a linkage group (2.6±1.2 vs 1.8±1.0, *P* = 0.020), Data was shown in Table 5 and Fig 1C. The correlation of previous defined linkages of gO2b/gN2, gO4/gN4b and viral load were not analyzed because of the limited sample numbers.

**Table 5:** Association between gO/gN genotype linkages and viral load.

	gO1a/gN4a(n)*			gO1a/gN1(n) *			gO3/gN4a(n) *		
	Presence (16)	Absence^#^ (34)	*P* Value	Presence (9)	Absence^#^ (41)	*P* Value	Presence (9)	Absence^#^ (41)	*P* Value
Viral load (log10 copies/ml)	2.7(1.9–3.1)	2.0(1.3–2.6)	.020	2.4(0.7–2.9)	2.2(1.5–2.7)	NS	1.9(0.9–3.3)	2.3(1.6–2.7)	NS

* Values are given as median (interquartile range).

^#^ Absence of all the corresponding gO and gN genotypes.

NS: none significance.

## Discussion

In the present study on AIDS patients in Wuhan, the prevalence of HCMV DNA in blood samples was frequently found in up to 61.3%. And mixed HCMV infection with multiple glycoprotein (gB, gO and gN) genotypes was found in 71% of HCMV infected AIDS patients with CD4 count < 100/mm^**3**^. Previous studies demonstrated that HCMV viremia increases mortality in AIDS patients with low CD4 counts [25, 26]. In addition, multiple HCMV infection was correlated with lethal outcome of fetus during pregnancy [27], higher viral load, delayed virus clearance, and higher rate of virus recurrency after antiviral treatment in transplant recipients and AIDS patients [20, 28]. However, little is known with respect to what factors determine HCMV virulence in the time course of infection and the grade of the host immune status.

Most of the HCMV genome is conserved both in laboratory and clinical strains. However, encoding viral glycoprotein genes, such as gB, gH, gN and gO are known to be variable sequences and may play crucial roles in HCMV cell tropism and in elicitation of neutralizing antibodies by interaction with host’s immune system[4, 29]. Previous studies have shown that glycoprotein genes are quite stable during long term of infection course in hosts [30]. This implicates that certain polymorphisms might be associated with strain-specific features, such as tissue tropism, virulence, and HCMV-induced immunopathogenesis.

However, number of investigations about the relationship of glycoprotein genotypes and HCMV pathogenicity in AIDS patients showed controversial results. For instance, gB2 was observed being a predominant genotype in HIV/AIDS patients, but its association with HCMV retinitis yielding conflicting results [16, 31, 32]. The distribution of the gO genotypes in clinical isolates from different geographic regions and different disease settings demonstrated that no clear correlation exists between gO genotype and HCMV disease [20, 21, 33]. The gN4b was found associated with higher level of antigenemia in solid organ transplant recipients [34]. The gN1 and gN3a were shown associated with a more benign course of congenital HCMV infection and gN4 correlated to increased incidence of sequelae [35]. However, gN genotyping analysis evaluated in a large number of adult SOT recipients and hematopoietic stem cell transplant recipients showed that no single genotype of gN correlated with clinical features[36, 37]. Therefore, the effects of glycoprotein polymorphisms on pathogenesis of HCMV infection are largely unknown.

Since high HCMV viral load levels are significantly associated with death, independently of CD4 counts, other opportunistic infections and highly active antiretroviral therapy (HAARDT) in AIDS patients [25, 38, 39], Therefore, we choose viral load as indicator of HCMV virulence. For understanding the biological role of glycoprotein gene polymorphisms for HCMV virulence, the current study investigated the association between the distribution of gB, gO, gN genotypes and the viral load in blood. Our study clearly demonstrated that the genotype gB1 was predominant and composed 86.96% of patients. This is in contrast to the predominant distribution of gB2 in AIDS patients and may associate with higher viral load and retinitis [11, 31, 32]. gO1a and gN4a were the other two predominant genotypes in our cohort, composed 78.8% and 49.2% of patients, respectively. Although gB1 and gO1a were predominant genotypes, they do not have an impact on HCMV viral load compared to other gB and gO genotypes. Interestingly, gN4a is significantly associated with higher-level of viral load (*P* = 0.048), which suggests that gN4a may play a role in virulence of HCMV in AIDS patients.

Based on the discrepancy with regard to the distribution of gB, gO and gN genotypes in different clinical settings and the virulence relation with certain gB, gO and gN genotypes in our study and in previous reports, we assume that virulence of HCMV may be dependent on synergistic action by at least two glycoproteins of HCMV. Previous studies illustrated no clear genetic linkages between gB and gN, gH and gO, gH and gN, gO and gH, gO and gL genotypes [7, 19, 20, 40], respectively. In contrast, the analysis of gO and gN genotypes in both laboratory strains and clinical isolates from congenitally infected newborns, transplant recipients and HIV/AIDS patients from Caucasian populations and Japanese infants illustrated that eight consistent linkages could be indentified between hypervariable gO and gN genotypes as follows: gO1a/gN1, gO1b/gN3a, gO1c/gN4c, gO2a/gN3b. gO2b/gN2, gO3/gN4a,gO4/gN4b, gO5/gN4c [21, 40]. Underlining the relationship of gO/gN linkages and HCMV disease, gO1a/gN1 was observed initially having an association with HCMV disease in HIV/AIDS patients and in congenital infections [19, 41]. Therefore, the linkage of gO/gN genotypes may have a potential functional interaction between these glycoproteins with their corresponding complexes, gC-II (gM/gN) and gC-III (gH/gL/gO). These complexes play a role in attachment, replication and spread of HCMV.

In summary, in the present study a novel potential linkage of gO1a/gN4a was identified. The gO1a/gN4a was the most frequent detected glycoprotein linkage (51.5%) in HCMV infected AIDS patients and was significantly associated with higher level of viral load compared to other defined gO/gN linkages, despite gO1a alone didn’t correlate to the level of viral load. The phenotype of a gO deletion mutant reveals that gO plays a critical role in secondary envelopment and is required for cell-to-cell transmission in fibroblasts [42]. The gO can bind to gH/gL forming gH/gL/gO complex [43, 44] which promotes the virus entry into various cell types [45]. Concerning the previous studies about the function of gO, our analyses imply that the genetic linkage between gO1a and gN4a may have a functional interaction, which possible contribute to virus replication, entry and cell tropism. The synergistic action by gO and gN, specifically by gO1a and gN4a may play an important role in virulence of HCMV. Therefore, the relationship of gO1a /gN4a and HCMV disease in AIDS patients should be taken into account. In contrast to the previous studies [19, 41], no association was observed between gO1a/gN1, gO3/gN4a and viral load, which may be due to limited sample numbers in this study.

Up to now, we don’t really understand how different HCMV strains interact with each other and what the biological significance of this interaction could be, particularly in immunedeficient patients, even though mixed HCMV strain infection showed disadvantageous for these patients. Either the formation of matched gO/gN genotypes due to recombinant virus induced by recombination of glycoprotein genes between two virus strains or due to interactions by different strains during mixed infection, is an important question. Several previous studies analyzed sequences from HCMV clinical isolates and laboratory-adapted strains and showed that genomic rearrangements and deletions occurred for giving added functions to immunomodulation or infectivity of HCMV [46–48]. In addition, the location between the gO and gN genes is a major site for rearrangements between herpesvirus lineages [49]. It suggests that some recombinations may occur between gO/gN sub-groupings in the infected host. However, perivous study in the murine model infected with two distinct MCMV strains, demonstrated that coinfection just alters virus fitness by functional trans-complementation rather than by genetic recombination [50]. Therefore, how synergistic contribution of variants which enhance HCMV pathogenicity in the multiple HCMV infected host, needs further investigation.

## Conclusion

The multiple HCMV strain infection is quite frequent in advanced AIDS patients. Although the genotype gB1, gO1a, and gN4a of HCMV are the predominant glycoprotein genotypes infection, only gN4a is correlated to higher level of viral load. Moreover, a novel gO1a/gN4a linkage is identified, which is associated with an increased viral load. Therefore, the pathogenic mechanisms underlying glycoprotein polymorphisms and the interaction of virus variants are needed to further study.

## Supporting information

S1 Table Viral load of all patients with respective genotypes. This is the raw data file for all analyses.
(DOC)
