# Supplementary material for: Human cytomegalovirus glycoprotein polymorphisms and increasing viral load in AIDS patients
Source: PLoS One. 2017 May 3;12(5):e0176160. doi: 10.1371/journal.pone.0176160 (PMC5415198; doi:10.1371/journal.pone.0176160)
Supplement: S1 Table — This is the raw data file for all analyses. (DOC) [file pone.0176160.s001.doc]

**Viral load of all patients with respective genotypes**

| **Sample**  **No.** | **HCMV DNA load** | | **GB** | | | | | **gN** | | | | | | | **gO** | | | | | | | |
| --- | --- | --- | --- | --- | --- | --- | --- | --- | --- | --- | --- | --- | --- | --- | --- | --- | --- | --- | --- | --- | --- | --- |
|  |  |  | **gB1** | **gB2** | **gB3** | **gB4** | **gB5** | **gN1** | **gN2** | **gN3a** | **gN3b** | **gN4a** | **gN4b** | **gN4c** | **gO1a** | **gO1b** | **gO1c** | **gO2a** | **gO2b** | **gO3** | **gO4** | **gO5** |
|  | **(copys/ml)** | **(log10)(copies/ml)** |  |  |  |  |  |  |  |  |  |  |  |  |  |  |  |  |  |  |  |  |
| **6** | **3140.871** | **3.5** | **+** |  |  |  | **+** |  | **+** |  | **+** |  |  |  |  |  |  |  | **+** |  |  |  |
| **7** |  |  |  |  |  |  |  |  |  |  |  |  |  |  |  |  |  |  |  |  |  |  |
| **45** |  |  | **+** | **+** |  |  | **+** | **+** | **+** |  | **+** |  |  |  | **+** |  |  |  |  | **+** |  |  |
| **58** | **1.410094** | **0.17** | **+** | **+** |  |  | **+** | **+** |  |  |  |  |  |  | **+** |  | **+** |  | **+** | **+** |  |  |
| **92** |  |  |  |  |  |  |  |  |  |  |  |  |  |  |  |  |  |  |  |  |  |  |
| **72** | **16.14945** | **0.15** | **+** |  |  |  | **+** | **+** |  |  |  | **+** |  |  | **+** |  |  |  | **+** | **+** |  |  |
| **73** | **3.70812** | **0.51** | **+** | **+** |  |  | **+** |  | **+** |  |  | **+** |  |  | **+** |  | **+** |  |  | **+** |  |  |
| **93** | **18.4626** |  |  |  |  |  |  | **+** | **+** |  | **+** |  |  |  |  |  |  |  |  |  |  |  |
| **94** | **36.75432** | **0.57** |  |  |  |  |  |  | **+** |  |  |  |  |  |  |  |  |  |  |  |  |  |
| **95** | **1.20856** | **1.27** | **+** |  |  |  |  | **+** |  |  |  |  |  |  |  |  |  |  |  |  |  |  |
| **99** | **0.27791** | **1.57** |  |  |  |  |  |  |  |  | **+** |  |  | **+** |  |  |  |  |  | **+** |  |  |
| **103** | **1.07249** |  |  |  |  |  |  |  |  |  |  |  |  | **+** |  |  |  |  |  |  |  |  |
| **107** | **2.69387** |  |  |  |  |  |  |  | **+** |  |  |  |  |  |  |  |  |  |  |  |  |  |
| **108** | **4.78956** | **1.28** | **+** | **+** | **+** |  |  |  | **+** |  |  | **+** |  |  |  |  | **+** |  | **+** | **+** |  |  |
| **112** | **4.413** |  | **+** | **+** | **+** |  |  |  |  |  |  |  |  |  |  |  |  |  |  |  |  |  |
| **210** |  |  |  |  |  |  |  |  |  |  |  |  |  |  |  |  |  |  |  |  |  |  |
| **347** | **290.57** | **0.68** | **+** | **+** | **+** |  |  |  | **+** |  |  | **+** | **+** |  |  |  |  |  |  |  |  |  |
| **435** | **34.56275** | **0.64** | **+** |  | **+** |  |  |  |  |  | **+** |  |  |  |  |  |  |  |  |  |  |  |
| **444** | **3391.823** |  | **+** | **+** | **+** |  |  |  | **+** |  |  | **+** |  |  |  |  |  |  |  |  |  |  |
| **445** | **481.5502** |  | **+** |  | **+** |  |  |  | **+** |  |  | **+** |  |  | **+** |  |  |  |  |  |  |  |
| **446** | **727.8055** | **1.54** | **+** |  | **+** |  |  |  |  |  |  | **+** |  |  |  |  |  |  |  |  |  |  |
| **480** | **255.7936** | **3.53** | **+** |  | **+** |  |  |  | **+** |  |  | **+** |  |  | **+** |  |  |  |  | **+** |  | **+** |
| **481** | **97.34776** | **2.68** |  | **+** | **+** | **+** |  |  |  |  | **+** |  |  |  |  |  |  |  |  |  |  |  |
| **482** | **943.239** | **2.86** | **+** |  | **+** | **+** |  |  | **+** |  |  | **+** |  |  |  |  |  |  |  |  |  |  |
| **501** | **54.33889** | **2.41** | **+** |  |  |  |  |  |  |  |  | **+** | **+** |  | **+** |  |  |  |  |  |  |  |
| **503** | **275.6226** | **1.99** | **+** |  |  |  |  |  |  |  |  |  | **+** |  | **+** |  |  |  |  |  |  |  |
| **506** | **417.4447** | **2.97** | **+** |  |  |  |  | **+** | **+** |  |  |  |  | **+** | **+** |  |  |  |  |  |  |  |
| **507** | **71.40944** | **1.74** | **+** |  | **+** |  |  |  | **+** |  |  | **+** |  |  | **+** |  |  |  | **+** |  |  |  |
| **508** | **22.03466** | **2.44** | **+** |  | **+** | **+** |  | **+** | **+** |  |  | **+** |  |  | **+** |  |  |  |  |  |  |  |
| **509** | **1344.613** | **2.62** |  |  |  |  |  |  |  |  |  | **+** |  |  |  |  |  |  |  |  |  |  |
| **510** | **971.647** |  | **+** |  |  |  |  |  | **+** |  |  |  |  |  |  |  |  |  |  |  |  |  |
| **511** | **209.0345** | **1.34** | **+** |  |  |  |  |  | **+** |  |  | **+** |  |  |  |  | **+** |  |  | **+** |  |  |
| **512** | **356.254** |  |  |  |  |  |  | **+** |  |  |  |  |  |  |  |  |  |  |  |  |  |  |
| **515** | **1232.957** |  | **+** |  | **+** |  |  |  | **+** |  |  |  |  |  |  |  |  |  |  |  |  |  |
| **517** | **85.76467** | **2.32** |  |  | **+** | **+** |  |  |  |  |  | **+** |  |  | **+** |  |  |  |  |  |  | **+** |
| **519** | **1123.094** | **2.55** |  |  |  |  |  |  | **+** |  |  | **+** |  |  |  |  |  |  |  |  |  |  |
| **520** | **798.14** | **3.09** | **+** |  | **+** | **+** |  |  |  |  |  | **+** |  |  | **+** |  |  | **+** |  | **+** |  |  |
| **525** | **436.0837** | **1.93** |  |  | **+** | **+** |  |  | **+** | **+** |  | **+** |  |  |  | **+** |  | **+** |  | **+** |  | **+** |
| **526** | **1123.439** | **3.05** |  |  |  |  | **+** |  |  | **+** |  | **+** |  |  |  |  |  |  |  |  |  |  |
| **531** | **146.8426** | **2.9** |  |  | **+** | **+** |  |  |  |  |  | **+** |  |  | **+** |  |  |  |  |  |  |  |
| **534** | **209.2658** | **2.64** | **+** |  | **+** |  |  |  | **+** | **+** |  |  |  |  | **+** |  | **+** |  |  |  |  |  |
| **535** | **136.0409** | **3.05** | **+** |  | **+** |  |  |  |  |  |  | **+** |  |  | **+** |  |  | **+** |  |  |  |  |
| **537** | **0.052608** | **2.17** |  |  | **+** |  |  |  |  |  | **+** |  |  |  | **+** |  |  |  |  |  |  |  |
| **538** | **78558.09** | **2.32** | **+** |  |  |  |  | **+** |  |  |  | **+** |  |  | **+** |  |  |  |  |  | **+** |  |
| **539** | **887.4607** | **2.32** | **+** |  | **+** |  |  |  |  |  |  | **+** |  |  |  |  |  |  |  |  |  |  |
| **542** | **81.50679** | **-1.28** |  |  |  |  |  | **+** |  |  | **+** |  |  |  |  |  |  | **+** | **+** |  |  |  |
| **543** | **298.4752** | **4.9** | **+** |  | **+** | **+** |  |  | **+** |  |  | **+** |  |  | **+** |  |  |  |  | **+** |  | **+** |
| **544** | **267.0809** | **2.95** | **+** |  | **+** |  |  | **+** | **+** |  |  | **+** |  |  | **+** |  |  |  |  |  |  |  |
| **545** | **656.1346** | **1.91** | **+** |  | **+** |  |  |  | **+** |  |  |  |  |  | **+** |  |  |  |  |  |  |  |
| **546** | **124.3114** | **2.47** | **+** |  |  |  |  | **+** |  |  |  |  |  |  | **+** |  |  |  |  |  |  |  |
| **547** | **2.72334** | **2.43** |  |  |  |  |  |  |  | **+** |  |  |  |  |  |  |  |  |  |  |  |  |
| **549** | **852.4693** | **2.82** | **+** |  |  |  |  | **+** |  |  |  |  |  |  |  |  |  |  |  |  |  |  |
| **550** | **74.89907** | **2.09** |  |  |  |  |  |  |  |  |  | **+** |  |  |  |  |  |  | **+** | **+** |  |  |
| **551** | **15.08927** | **0.43** |  |  |  |  |  | **+** |  |  |  |  |  |  |  |  |  |  |  |  |  |  |
| **567** | **60.66228** | **2.93** | **+** |  | **+** |  |  | **+** |  |  |  |  |  |  | **+** |  |  |  |  |  |  |  |
| **577** | **6580.741** | **1.87** | **+** |  |  |  |  | **+** |  |  | **+** |  | **+** |  |  |  |  |  |  |  |  |  |
| **581** |  |  |  |  |  |  |  |  |  |  |  |  |  |  |  |  |  |  |  |  |  |  |
| **583** | **175.3643** | **1.78** | **+** |  | **+** |  |  |  | **+** |  |  | **+** |  |  | **+** |  |  |  |  |  |  |  |
| **587** | **150.0977** | **3.82** | **+** | **+** | **+** |  |  |  |  |  |  | **+** | **+** |  | **+** |  |  |  | **+** |  | **+** |  |
| **590** | **175.36**  **1** | **2.24** |  |  |  |  |  |  |  |  |  |  |  |  |  |  |  |  |  |  |  |  |
| **591** | **29.07585** | **2.18** |  |  |  |  |  |  |  |  | **+** |  |  |  |  |  |  |  |  |  |  |  |
| **592** | **14.47363** |  | **+** |  |  |  |  |  |  |  | **+** |  |  |  |  |  |  |  |  |  |  |  |
| **600** | **3140.871** | **1.81** | **+** |  |  |  |  |  |  |  | **+** |  |  |  |  |  |  |  |  |  |  |  |
| **601** | **3.1274** | **1.46** |  |  |  |  |  |  |  |  | **+** |  |  |  |  |  |  |  |  |  |  |  |
| **605** | **1.476071** | **1.16** | **+** |  | **+** |  |  | **+** | **+** |  | **+** |  |  |  | **+** |  |  |  |  | **+** |  |  |
